# Supplementary material for: Proposed standards for prosthetic foot reuse and considerations for donation of used prosthetic feet to low-and middle-income countries
Source: PLOS Glob Public Health. 2026 May 13;6(5):e0006270. doi: 10.1371/journal.pgph.0006270 (PMC13170859; doi:10.1371/journal.pgph.0006270)
Supplement: S1 File — (DOCX) [file pgph.0006270.s002.docx]

Model diagnostics for model m7:

The binned residual plot (Figure 1) was used to determine if there were systematic misfits. As all points are near zero, this implies the model’s predicted probabilities are close, on average, to the observed outcomes. The random scatter of the datapoints suggests the logit link function is appropriate and there are no missing nonlinear or interaction parameters.


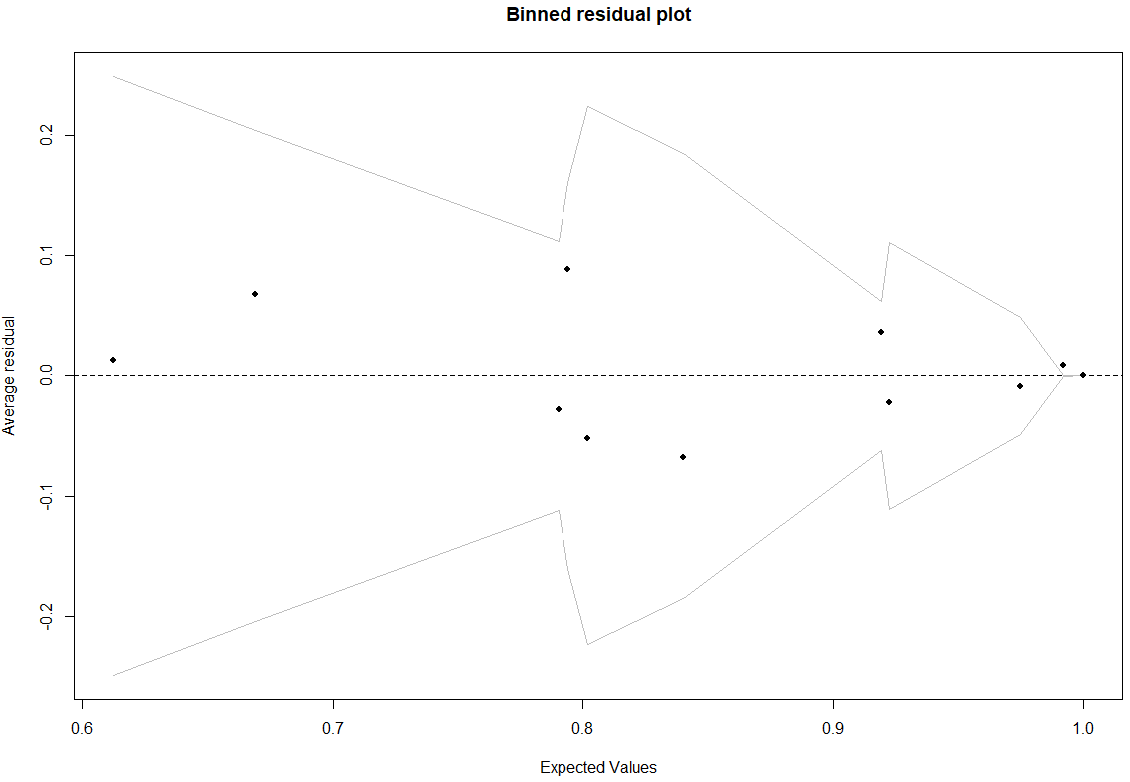


Figure 1: Binned residual plot.

The original Cook’s distance plot showed “BioQuest” (n = 1) to have an exceedingly high value (e20). This is because brand was treated as a factor in R, and R measured the coefficients for brand relative to the first brand, alphabetically, which was BioQuest. The small sample for the reference brand (n = 1) made the model unstable. When BioQuest was recoded as “zzz”, enabling Blatchfords (n = 120) to be the reference brand, the Cook’s distance plot stabilised demonstrating the statistical model was stable (Figure 2).


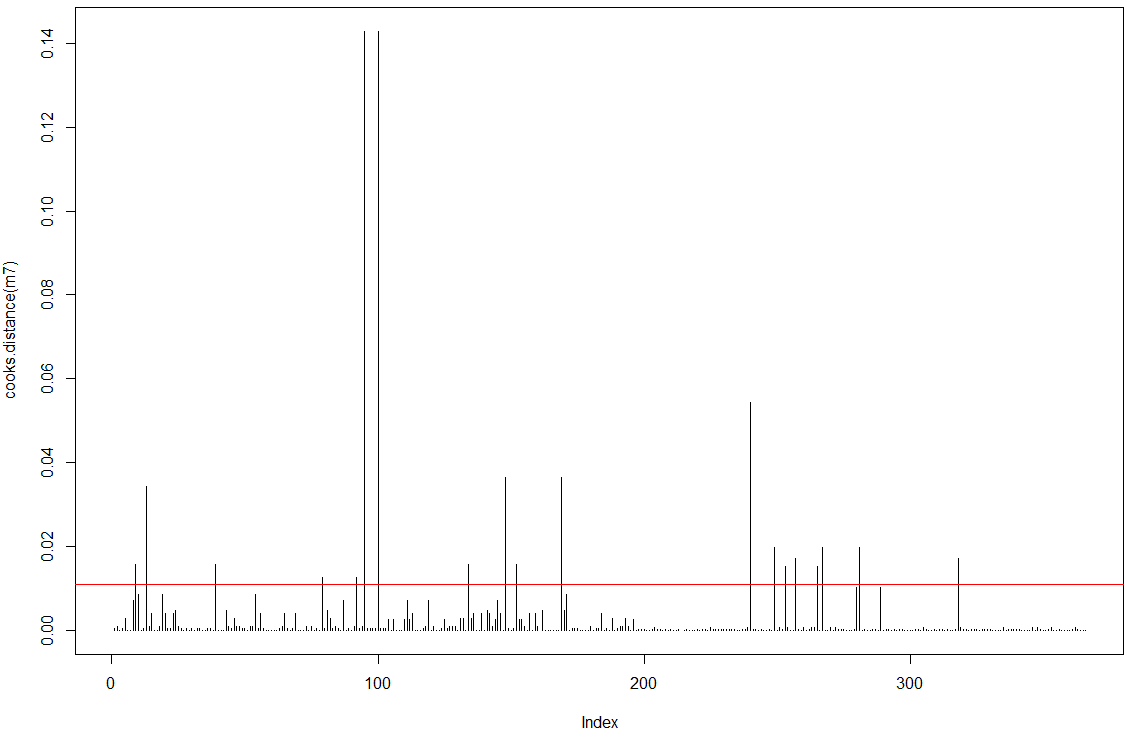


Figure 2: Cook's distance plot.

The Hosmer-Lemeshow goodness-of-fit test ($\chi^{2}=2.2833$, df = 4, p = 0.8919) demonstrates minimal discrepancy between predicted and observed values, demonstrating the model was well-calibrated and there is no indication of missing structure (e.g., interaction or nonlinear terms). Overdispersion (0.6242) indicates slight underdispersion in the model. The area under the curve (auc = 0.7852) indicates good discriminatory ability in the model and that the model is not overfit.
