# Supplementary material for: Proposed standards for prosthetic foot reuse and considerations for donation of used prosthetic feet to low-and middle-income countries
Source: PLOS Glob Public Health. 2026 May 13;6(5):e0006270. doi: 10.1371/journal.pgph.0006270 (PMC13170859; doi:10.1371/journal.pgph.0006270)
Supplement: Attachment — Submitted filename: Response to reviewers.docx [file pgph.0006270.s004.docx]

Comments to the Author

1. Does this manuscript meet PLOS Global Public Health’s publication criteria? Is the manuscript technically sound, and do the data support the conclusions? The manuscript must describe methodologically and ethically rigorous research with conclusions that are appropriately drawn based on the data presented.

Reviewer #1: No

Reviewer #2: Yes

Reviewer #3: Partly

2. Has the statistical analysis been performed appropriately and rigorously?

Reviewer #1: No

Reviewer #2: Yes

Reviewer #3: Yes

3. Have the authors made all data underlying the findings in their manuscript fully available (please refer to the Data Availability Statement at the start of the manuscript PDF file)?

Reviewer #1: Yes

Reviewer #2: Yes

Reviewer #3: Yes

4. Is the manuscript presented in an intelligible fashion and written in standard English?

Reviewer #1: No

Reviewer #2: Yes

Reviewer #3: Yes

5. Review Comments to the Author

Reviewer #1: 1. The manuscript primarily shows that adding a visual inspection step increased the proportion of prosthetic feet deemed usable (83% to 94%). This outcome is predictable and does not constitute meaningful scientific innovation. The work reads as an operational description rather than rigorous research; novelty and contribution are therefore limited.

We thank the reviewer for taking the time to review the manuscript and helping us improve this resubmission.

2. The proposed checklist is not validated. There is no mechanical or structural testing, no clinical functional outcomes, no prospective field evaluation, no inter-rater reliability assessment, and no sensitivity or specificity analysis. Accordingly, the checklist cannot be considered a standard, and the conclusions overstate the evidence. A formal validation phase is required.

Most of this comment is addressed below, but in brief, this method was evaluated “in the field”. We propose feet must be checked before getting to Africa (or place of use), and that was done in this study. The feet were also “re-checked” in the field, in Uganda.

As discussed in more detail in response to Comments 2-4, we accept that the checklist is not a standard and hence have revised the title of the paper. The new title is ”Towards standards for prosthetic reuse: considerations for donation of used prosthetic feet to low- and middle-income settings”.

3. Safety, mechanical integrity, and lifespan have not been evaluated. Visual inspection alone is inadequate for medical devices. No ISO-aligned static or cyclic loading tests are presented, nor are durability or time-in-service data available. This is a critical omission given the manuscript’s intent to inform international practice.

4. No patient-level outcomes are included (for example, fit success, comfort, skin issues, mobility, abandonment, repair frequency, or time-to-failure). Without these data, the practical value of the intervention remains uncertain.

Comments 2-4: We agree this method should be tested and retested in numerous manners, including those mentioned by the reviewer. We have modified the language in the manuscript to reflect that, before the checklist we have proposed be taken on as a set of international standards, they must be rigorously tested. We have also expanded on these concerns in the “Future Work” section. While we agree this work must be carried out, we also believe a set of standards must first be proposed in some form so that they can be tested and altered as needed.

We also recognize the limitation of a visual inspection. Visual inspections alone are sometimes adequate for the provision of medical devices, e.g., inspecting packaging to ensure orthopaedic devices are still sterile. We also believe some type of ISO-aligned mechanical tests may be appropriate and have taken the following exert from the “Future Work” section of the original submission.

To begin this journey, we proposed a set of standards in the form of a quality assurance check that focus on prosthetic foot quality at the point of provision (Table 5). However, these standards do not test product safety or lifespan. Rapid, affordable, and non-/minimally destructive tests should be created to test prosthetic foot safety/lifespan to promote prosthetic user safety and ensure users will not need to have their devices repaired/replaced soon after receipt. Some test(s) should focus on the structural integrity of the keel, potentially similarly to the ISO 10328 and 22675 tests.

5. Brand-level comparisons are underpowered, and model-level or material-level analyses are not presented. Despite acknowledging this limitation, the manuscript still interprets brand-related effects.

To conduct a power analysis for the effect of brand in a binomial glm, we must first define what we mean by power. Some ways we could do this is by asking:

1) What is the global effect of brand? i.e., do any of the 17 brands differ from a single reference brand?
2) What is the power for individual brand contrasts? i.e., do I have the power to detect the difference between brand X and brand Y?

The first question can be addressed with a likelihood ratio test, comparing m7 to m2 (i.e., comparing the statistical best model to the best statistical model that does not include brand). When we do this using the code anova(m7,m2,test="LRT") in R, we get a p-value of 0.0006641 indicating that there is a strong effect of brand. We could also test this using a Wald chi-squared test using the code drop1(m7, test = "Chisq") and get an identical answer. These results have now been reported in the manuscript.

We did not address question 2 in our manuscript – while brand-level differences likely exist, it is impossible to isolate the effect of brand from confounding factors, such as P&O centre from which the foot originates. As we cannot tease apart these effects, we have not reported on the results of the second analysis in our manuscript. We believe this helps keep our results focused.

6. The Introduction and narrative sections are disproportionately long and repetitive; substantial condensation is recommended. In contrast, the Methods and Results require greater depth and clarity.

We agree that, proportionally, the narrative sections are long compared to the Methods and Results. This is something we, the authors, discussed prior to submission. This approach is more in line with studies in the social sciences, particularly with the relatively high number of quotes. We ultimately felt these longer narrative sections were needed to review what has been done, as there is a paucity of literature on the donation model for prosthetic provision, and we could not find any published material which summarised this work. This helped us interpret our results in the larger framework. We felt the M&M and Results sections did not require as much space to fully dictate what we did, and lengthening these sections was not necessary for the message of the paper. We have, however, expanded these sections as necessary to add new analyses/results.

7. The statistical analysis is limited. Logistic models do not account for key confounders such as service age, storage duration, materials, or model type. Model diagnostics, effect sizes with confidence intervals, and multiple-comparison considerations are not reported.

The generalised linear models used in this study took model type into account. Factors such as service age, storage duration, and materials could not be considered in the statistical models, as it was not possible to gather this data in the field (although, as stated in the M&M, we do know the NCRPPD feet were 1+ years old, while the K4C feet were <1 year old). We have now investigated effect size of brand and included in the text. It is not standard to report on confidence intervals for AIC/dAIC/Weights or the coefficients in Table 4 as these are deterministic values.

When using AIC to determine which statistical model best fits the data, it is not standard to report on model diagnostics, but we have now included some diagnostic tests in the ESM and summarized the findings below.

Briefly, the Cooks Distance for brand BioQuest was abnormally high – this was because it was, alphabetically, the first brand listed. As such, all other brands were measured relative to BioQuest, which had a sample of n = 1. By changing the name “BioQuest” to “zzz”, thus allowing “Blatchfords”, which had a larger sample, to be listed first alphabetically, all Cooks Distances became relatively small, and the model stabilized. This caused the numeric values in Table 4 to change, as brands were no longer being measured relative to BioQuest, but the results from the study remained unchanged.

When this is done, our model diagnostics conclude there are no systematic misfits in the data, no extremely influential points, that the model has excellent calibration, no overdispersion, and good discrimination, meaning it is good for predictive use.

Despite indication in our model diagnostics that we were not missing out on interaction parameters, To gain further confidence in our statistical model, and that it was the best fit for our data, we used the “dredge” function in MuMin to investigate interaction effects as well. The four best statistical models had dAIC (delta) values less than 0.26, indicating they all performed about the same. Together, they had a cumulative weight of 0.847, and the fifth best model had a delta >2 with a weight ~1/3 of the previous model, indicating it did not perform as well.

The four best models (below) agreed that brand (Brn) and site (Sit) should be included in any statistical models, and side (Sid) and size (Siz), along with most interaction parameters, should be excluded. The models disagreed on whether model (Mdl) or the interaction effects between brand and model or model and site should be included. Given the increased complexity of models with interaction parameters and risk of overfitting the data, we chose to go with model 10 in the table below (m7 in our paper), as this was the best model that did not include interaction effects.

8. Economic evaluation is absent. Donation and reuse programs in low and middle income settings are cost sensitive, and without cost modeling, the recommendations have limited actionable value.

See response to points 2-4.

9. Several claims are overstated, including suggestions related to circular economy effects, international standard development, and safety assurance. These assertions are not supported by the presented data and should be moderated.

We have moderated our language throughout the manuscript.

Reviewer #2: It is suggested to review the Nippon Foundation/Exceed Cambodia in proposing the standards of P&O. The case study that has been done in Cambodia, Myanmar, Laos, Vietnam and Sri Lanka in will guide the current P&O Standard in low and middle income countries.

It is best to review the minimum standards of P&O in these countries as a underlying theory to govern the foundation of foot reuse and donation used.

A robust systematic reviews are vital in proposing standards for foot reuse and donations used in low and middle income countries. An updated literature are needed.

It is suggested to explore the preliminary findings in these low and middle income countries.

We thank the reviewer for taking the time to review the manuscript and provide their invaluable input and support. We have tempered the language surrounding the proposal of standards – as mentioned in our future work and limitations sections, we do not expect this paper to be an “end-all” for prosthetic foot donation, but rather a step towards the development of standards. As such, while we conducted a comprehensive review at the time of writing, a systematic review goes beyond the limitations of this study. We hope that, at such time as international standards are developed (e.g., by ISPO or the WHO), a relevant systamtic review would be conducted by the leading organisation.

With regard to the Nippon Foundation/Exceed Cambodia, to our knowledge, they focus on the comprehensive provision of prosthetics in LMICs, focussing on SE Asia. By engaging with multilevel stakeholders, they enable the construction/development of P&O schools, supply chains, and provision throughout the country. Some of us (the authors) are part of the Exceed Research Network and – to our knowledge – Exceed has not engaged with the donation of prosthetics, but rather works towards the development of affordable, locally relevant prosthetics. Members of Exceed have been involved in some work investigating the donation of prosthetics, (e.g., Ghidini et al., 2025 <https://doi.org/10.1080/09638288.2025.2509792>; the work in collaboration with the University of Southampton investigating the donation Jaipur prosthetics) but this has focused on whether prosthetics *should* be donated rather than *how* to safely donate prosthetic components/devices.

We reached out to Exceed, through the ERN, shared this comment, and asked about their work with the Nippon Foundation and standards related to donation of prosthetics, or provision of prosthetics, in general, in LMICs. They shared their 100+ page report, but were unsure how it related to the reviewer’s comments, and as such we were unable to address this point or include this information in our manuscript.

We agree there are certainly drawbacks to the donation of prosthetic components/devices. However, despite these issues, prosthetic devices will continue to be donated. It is because of this that we wish to begin to investigate what would make a prosthetic fit to be donated. Ghidini et al., 2025, for example, looked at donated modular vs locally manufactured components, and found the locally manufactured ones to be more durable. Studies like this are important for understanding if prosthetic components should be donated, but what we want to do in this study is begin to understand how we could determine if prosthetics are safe for donation. Ultimately, both are important and related (e.g., if ISPO or the WHO were to create standards for donating prosthetics) but a review of whether prosthetics *should* be donated is beyond the scope of this study.

Reviewer #3: GENERAL

This reviewer welcomes the ambition of the authors to start developing standards for donated prosthetic componentry to LMICs. Such standards are indeed much needed as one important factor to improve the quality of the prosthetic devices provided within LMICs. The authors’ work has carefully been imbedded into a wealth of information and reasons for why the need is urgent for developing standards of donated prosthetic components. This information has been mindfully drafted including viewpoints and situation of many LMICs as well as HICs. Well done!

We thank the reviewer for her kind comments and taking the time to provide such a detailed and helpful review, which has certainly improved the manuscript substantially!

What left this reviewer wondering is why the development of the checklist has not been carried out with locals at the two centers, where MB and PM were able to collect the data of the stored feet. The rationale for not doing so should be included into the Limitations section.

We agree the inclusion of local collaborators is important when conducting work overseas. Louise Ackers in the director of K4C and lives many months of the year at her house in Uganda, and we believed it was appropriate for her to fulfil this role. We have listed relevant information in the Competing Interests section.

Further, why has no testing of the developed checklist been carried out with the two centers? For example, dividing the available feet into two equal sized groups would have raised the opportunity to develop the checklist with one group of feet including the regression model and then test it on the remaining feet in the second group. Why was this not considered? One could classify all available feet as indicated in Table 1, but then consider only these feet who were mostly used in the field or were mostly available. Lowering the numbers of independent variables to the those variables that would represent the essence of the checklist best would have given the option for a regression model, or is this reviewer mistaken? These points should be discussed in the paper. In case the paper gets too long (word count), it is recommended to concise the actual discussion section as it provides similar points stated in the introduction.

Apologies if we have misinterpreted the reviewer’s comment in this response. We believe the reviewer is asking why we did not take a sample of ~400 feet in Uganda, divide the sample in 2, and use half the feet to develop a checklist. We then would use that group of ~200 feet to build a regression model to determine which factors were important in determining whether a foot was usable or not, and then test this regression model, validating it, with the second group of feet.

If so, this is a valid, interesting, and useful approach. However, we believe this approach would be more appropriate for the question, “Which factors affect foot usability?” rather than ours, which was, “What is the efficacy of this already developed checklist?” All that was necessary for this to be true was that the parameter “site” be statistically significant. We have added a paragraph to the “Future Work” section reflecting the need for additional work to understand which prosthetics are better/worse for donations.

And lastly, this reviewer does not think that retesting used feet similar to the stated ISO standards would be feasible. Instead, it might be worthwhile checking in other industries (aviation, deep-sea shipping) what type of non-mechanical controls for checking of wear and tear on materials/motors are available without dismantling motors or testing of used structures. Perhaps some light and/or sonar evaluation would be a way to check the mechanical structure of used prosthetic feet and other componentry without putting any more strain on the used materials. That might be some thoughts for the Future Work section. Also probable collaboration with universities in LMICs should be considered as a close source of additional brain power for the development of standards within a given country.

Parts of the author team are currently doing just this 😊 quickly, and without revealing much (as funds are being applied for), it is possible to use sound waves to identify cracks in materials, which is what is done in metals (like what was alluded to here). Fillauer tried to apply ultrasound in this manner and was unsuccessful due to difficulties with using ultrasound to identify cracks in carbon fibre composites. After talking with ~10 manufacturers at ISPO in July 2025, they believed an altered version of the ISO standards would be appropriate, and the lead author is currently working with Ossür towards this, but the project(s) are still being set up.

DETAILED

The reviewer finds the word ‘prosthetics’ difficult and prefers the (correct) term ‘prosthetic componentry or prosthetic components’ instead. In her experience using the nomenclature of the P/O profession adds clarity in an interdisciplinary context. It is often unclear to people outside of or adjacent to the P/O profession that a ‘prosthetics’ is composed of different products, i.e. some industrial produced prosthetic components and – in most cases – a bespoken locally fabricated prosthetic socket. By using prosthetic components or prosthesis/prostheses when referring to the final product – the authors will signal directly that there are ‘pieces’ needed to compose an entire prosthesis. Further, using the correct term assists in distinguishing prostheses fabricated with componentry from those being fabricated by 3D printing, also a field needing standards for C2C design. Therefore, please change the wording accordingly within the entire paper – thank you!

We thank the reviewer for catching this error and aiding in our interdisciplinary communication. We have gone through and added “component” where appropriate to improve our communication.

Lines 165-168. This sentence seems to be incomplete – please check.

Changed.

Line 229. This statement is incorrect. In Switzerland (and the reviewer is sure this is the case in France, Netherlands and the UK), prosthetic componentry has different life/warranty cycles depending on the type of prosthetic component and its model. Please rephrase this sentence pointing out that different prosthetic components and their models have different life/warranty cycles set by the industrial manufacturers.

Changed.

Lines 284-286.This sentence is unclear: Are the authors checking prosthetic feet shipped to Africa prior to the study or as part of the study when these feet arrive in Africa? If they are analyzed prior to the study how do the authors make sure that the damage seen is indeed due to shipping and not due to storage, for example? If the authors controlled feet within the study time period, would the sentence not needed to be stated “… we review prosthetic feet ALSO in Africa.”? Or did the authors not review the feet at the study place, but only in Africa? Please clarify and rephrase – thank you. These clarifications/details seem to be better placed within the Materials and Methods Chapter.

We have clarified the sentence.

Lines 287-311, in particular lines 311-317. Because the authors use an experimental setup, variables are usually considered as ‘independent’ or ‘dependent’. Please clarify what variables (independent, dependent) were considered. All variables the authors used to classify the different feet need be listed together with the rationale for the decision to include them into the regression model, including their order.

Ok – are the variables listed on line 314 the once considered as independent variables to classify a prosthetic foot as ‘reusable’ or ‘not reusable’? If so, why? In other words, why do the authors consider the ‘brand’ to be more important than the condition of the foot itself? Or is it the case because only those feet that passed the visual test of being 'usable' were included into the regression model? Up to this point, this reviewer understood the aim of the study as being to develop a set of criteria to classify a prosthetic foot as reusable or not. If a visual pre-selection needs to be carried out first, how good/robust is the regression model that follows? Please clarify and add this clarification to the text – thank you.

The aim of the study is to test the efficacy of a checklist already developed by STAND (from the original submission, L280-83: “…we examine the efficacy of the organizational standards used by STAND, with the idea that proven efficacy of these standards could lay the groundwork for the development of future international standards.”). Ultimately, we altered it to reflect observations during data collection (“How could this be improved to catch the flaws in the feet at K4C, which were already checked so should, theoretically, be 100% usable?).

We have added a paragraph expanding on the inclusion of the different variables.

Lines 296-298. What variables (the authors call them ‘flaws’, if understood correctly) did the authors consider during the usability tests? How were these tests carried out?

A sentence has been added to the M&M.

What happened with the feet the authors did consider as ‘not usable’: where they removed from the total sample of 366 feet (see below remarks to line 319)?

If feet were removed, 100% of the feet would be usable! The feet were marked as “not usable” in the Excel sheet.

For illustration: assuming the authors used for their visual check a variable called ‘cracks within the cosmetic’: did the authors classify a foot as still usable when only surface cracks were available, or did they exclude any foot with a crack in its shell?

Any cracks in the foot shell made the foot unusable, as they indicate the foot shell is starting to degrade and breakdown.

What were the criteria to classify a SACH foot as ‘usable’? More detailed information about the entire method for the visual checks and the resulting classification needs to be stated.

A sentence has now been added. There were no specialty criteria for SACH feet – as with all feet, any cracking would cause a foot to be unusable for donation.

When did the authors add any of this variable into the regression model and they give some of the variables a weighting, i.e. were some of the variables considered more important than others, and if so, why? Please add this information and make a reference to Table 2 or better, create a new Table or flowchart showing the authors thoughts and decision process including the variables used upon which they based their decision to classify a foot as ‘usable’ or ‘not usable’. Clarification on this matter will strengthen the work as it helps the reader to better understand the authors’ rationale – thank you!

We did not weight variables, as the model is a form of linear regression. Assigning a weight would be akin to assigning a slope to a Pearson’s regression before you ran the regression analysis. Variables are weighted by the glm when they are assigned coefficients – coefficients with higher magnitudes have higher weightings, while those with lower coefficients have lower weightings.

The explanation was previously provided in the M&M section and has been altered for clarity.

Line 319. Please start the results section with “A total of 366 feet where analyzed, 196 left and 170 right feet…”

Changed.

Line 320. Please add “… and A brand could be identified for… ” – thank you.

Changed.

Lines 320-322. Based on the information given in Table 1, there were 12 brands identified as categories plus one category with feet unknown to the authors. Because ‘unknown’ is not a brand, the sentence needs to be rephrased – thank you.

Changed.

Lines 353-357. These sentences seem to be missing some text, at least, they do not make sense to this reviewer. In lines 353-355 the authors state that the feet of Trulife and Ossur performed worst. Then in the following lines the authors state that they are (nevertheless??) considered as appropriate for donation. Please clarify – thank you.

We have been careful to not state that one brand is appropriate for donation while another is not in this paper. We have clarified in the Results section that these results should not be used to determine which brands are appropriate for donation and which are not due to confounding factors that were not considered here.

Table 4. Please explain/add, either in the corresponding text (lines 350 and subsequently) how the negative signs have to be read. Why has the measurement made against ‘BioQuest’ and not ‘Janton’ and how do the authors explain the difference in the coefficient between these two feet? Both feet were represented with n=1, why is there a difference? Please explain and add the clarification into the text within the Discussion section – thank you.

Programmes like R list factors alphabetically, and measure values relative to this reference category. All other brand coefficients represent log-odds differences relative to that reference. In this case, the first brand, when brands are listed in alphabetical order, was BioQuest. Because of an issue that was later identified in the Cook’s distance plot when values were measured relative to BioQuest (due to its small sample size, see ESM), BioQuest was coded as “zBioQuest” allowing Blatchfords, with a larger sample size, to be listed first alphabetically. This solved the issue with the Cook’s distance plot but did not change the results.

The issues the reviewer has identified with differences in coefficients, despite having the sample size and percent of usable feet, is related to the coding of the glm function in R. Ultimately, these small differences are errors with parameter estimation, and this can be seen by looking at the 95% CI for the coefficients (presented below). Note Blatchfords is not listed – as this was the reference category, it does not have a coefficient and therefore does not have a confidence interval. To avoid a lengthy discussion about this in the paper, we have chosen not to present the 95% CIs. Rather, we hope this table shows that brand is an important factor to be considered for future studies and have more directly stated this in the Results and Future Work sections.

The caption of Table 4 has been altered defining, statistically, what the coefficients are.

Figure 2. Please add to Fig. 2, a, b, and c, as done in Fig. 1. This assists in clarifying matters. Please add this clarification into the text: line 364 = Figure 2a; line 378: delete (Figure 2) and add after ‘NCRPPD’ (Figure 2b); line 379: add (Figure 2c) after ‘K4C’.

Changed.

Line 388. Add at the end of the sentence ‘(Figure 3)’.

Changed.

Line 395. Please expand this sentence like or similar as proposed “…can be a burden to the recipient LMIC [31, 39,40], as indicated by Marks et al (2019 – Please check PLOS rules!!):” and then have the quotation followed. This will connect the quotation with the text and makes it easier to read.

While the quote is from Marks et al., 2019, we have not added, “as indicated by Marks et al XXX” as we believe this quote should be taken as fact and not attributed to a single study. We have cited Marks at the end of the quote.

Line 469. Please check this sentence – the word ‘design’ seems to be twice stated. If this is correct, consider rephrasing as the sentence reads strange, thank you.

Changed.

Checklist questions:

• Question (1): Please add example of ‘completeness’ of a prosthetic foot, as you did for Question 2.

Added.

• Question (3): Add examples of what the authors consider ‘compliant’: forefoot, heel, middle section? All of these, only one? Usable for light persons, like children if only one part of the foot is too compliant? If so, which one do the authors consider as the most important variable for a foot to be still considered ‘usable’?

We have not dictated a part of the foot as – when a polymer has begun to break down, delamination has occurred, the keel does not fit the foot shell appropriately, etc., – all parts of the foot will become more compliant. As this represents “whole foot failure”, we have not assigned differential importance to different parts of the foot, and would not advocate giving the device to a child.

Line 529. Word missing: “..cost of what” was the biggest barrier? Please complete.

Changed.

Line 533. Please consider replacing ‘in this way’ with ‘Therefore’ or similar that would connect clearer the content of the previous paragraph with this new one.

Changed.

Line 544. Typos: ‘reduce’ instead of ‘reduces’, ‘limit’ instead of ‘limits’.

Changed.

Line 567. Stop the sentence after ‘repair of equipment’ and continue with a new sentence starting, for example with “Hamner et al (please check PLOS rules!!) point out that … and than add the quotation.

Changed.

Line 570. Please delete ‘etc.’ This should not be used in a text as it lefts the reader wonder what else – in this case – could have had an influence. Instead write ‘for example’ and list the three most missing points that were not considered.

Both “for example” and “etc.,” are appropriate in this instance. We have decided to keep “etc.” to indicate to the reader that this is a non-exhaustive list and point them to paper [50] should they wish to read more about it.

Line 620. Keep the number correct: the authors tested 306 feet. The number speaks for itself, no need to bolster it. To this reviewer bolstering looks bad, stay with the figures.

Changed.

Line 622. Replace ‘are’ with ‘were’, as this was the case for the authors' sample. Samples of other authors might vary.

This sentence was written in the present tense. If this part of the sentence was changed to the past tense, other parts of the sentence (as well as the section) would need to be changed as well.

We would like to leave a final thank you to reviewer 3. It is difficult today to receive reviews, especially one that is so thoughtful and comprehensive. As such, we would like to give a giant “thank you!” for your efforts and positivity through the review. We hope we have changed the manuscript appropriately to reflect the input provided by the reviewer, which we believe has drastically improved the manuscript!
