# Supplementary material for: Proposed standards for prosthetic foot reuse and considerations for donation of used prosthetic feet to low-and middle-income countries
Source: PLOS Glob Public Health. 2026 May 13;6(5):e0006270. doi: 10.1371/journal.pgph.0006270 (PMC13170859; doi:10.1371/journal.pgph.0006270)
Supplement: Attachment — Submitted filename: Response_to_reviewers_auresp_2.docx [file pgph.0006270.s005.docx]

Reviewer #3: GENERAL
Congratulations to the authors for having completed the review. The amendments added to the manuscript have a large positive effect and strengthened it substantially. This reviewer also welcomes the clarifications added to the title: it captures in my point of view the essence of the manuscript. Excellent!

Thank you also for the big compliment I, as reviewer 3, have received. This was unexpected and much appreciated indeed!

Also appreciated is the authors’ response to my statistical question I had. Only some minor issues remain – please verify and adapt accordingly, thank you!

We thank the reviewer yet again for putting the time and effort into helping us improve the manuscript. As the reviewer has made their identity known, we have specifically thanked them in the Acknowledgements section!

DETAILED
Line 46: If word count allows, please spell out LMICs, thank you.

Changed.

Line 47: Missing full stop.

Changed.

Line 93: Thank you for the explanation given re ‘prosthetics’ and the changes you made throughout the manuscript. One seem to have been overlooked: please change ‘prosthetics’ to either ‘prostheses’, ‘prosthetic components’, ‘prosthetic provision’, ‘assistive devices’ or similar as it assists in clarifying matters – than you.

Apologies missed a few – changed.

Line 146: Please change ‘prosthetics’ to ‘prostheses’.

Changed.

Lines 235-38: Please change ‘prosthetics’ to ‘prostheses’ in all cases, as – according to this reviewers understanding, the authors are referring to an entire artificial device that replaces a limb. However, if you also refer to hearing aids or orthoses, then it is recommended to use the wider term ‘assistive devices’.

Changed.

Line 249: Please change ‘prosthetics’ to ‘prostheses’, as the authors refer to an entire prosthesis. If this is not the case, then delete the word ‘prosthetics’ as it would repeat the word ‘prosthetic components” – thank you!

Changed.

Line 303: a word seems to be missing: “(...) feet already AT/IN? prosthetic and orthotic centers (…).

Changed.

Line 482: Space missing between the words ‘permitted’ and ‘Ethical’.

Changed.

Line 508. Please delete the lonely full stop and adjust spacing – thank you.

Changed.

Line 542: please replace ‘prosthetics’ with either ‘prosthetic devices and componentry’ or ‘assistive devices and componentry’.

Changed.

Line 593: Please rephrase the start of this sentence to “Prosthetic feet can last long in LMICs (refs as stated) as they are overdesigned for their intended length of use (…) – thank you.

Changed.

Line 644: perhaps better “(…), device design is THE focus, (…)?

Changed.

Line 667: please delete ‘a’ so that it reads: “(…) proposed set of standards in (…)” – thank you.

Changed.

Lines 694-5: please add ‘for example’ after ‘is’ and replace ‘etc.,’ with ‘or other variables’ so that the line reads: “(…) usability is, for example, related to material, prosthetic component use, P&O centre policies/magnitude of use prior to donation, or other variables.” Thank you!

Changed.
